# Scale-up of the Physical Activity 4 Everyone (PA4E1) intervention in secondary schools: 12-month implementation outcomes from a cluster randomized controlled trial

**DOI:** 10.1186/s12966-020-01000-y

**Published:** 2020-08-08

**Authors:** Rachel Sutherland, Elizabeth Campbell, Matthew McLaughlin, Nicole Nathan, Luke Wolfenden, David R. Lubans, Philip J. Morgan, Karen Gillham, Chris Oldmeadow, Andrew Searles, Penny Reeves, Mandy Williams, Nicole Kajons, Andrew Bailey, James Boyer, Christophe Lecathelinais, Lynda Davies, Tom McKenzie, Jenna Hollis, John Wiggers

**Affiliations:** 1 Hunter New England Population Health, Locked Bag 10, Wallsend, NSW 2287 Australia; 2 School of Medicine and Public Health, University of Newcastle, Newcastle, 2308 Australia; 3 Hunter Medical Research Institute, Newcastle, NSW 2300 Australia; 4 Priority Research Centre in Physical Activity and Nutrition, School of Education, University of Newcastle, Newcastle, NSW Australia; 5 South Western Sydney Local Health District, Locked Mail Bag 7279, Liverpool BC, NSW 1871 Australia; 6 Central Coast Local Health District, 4-6 Watt Street, Gosford, NSW 2250 Australia; 7 Mid North Coast Local Health District, P.O. Box 126, Port Macquarie, NSW Australia; 8 New South Wales Department of Education, School Sports Unit, Level 3, 1 Oxford Street, Darlinghurst, NSW 2010 Australia

**Keywords:** Physical activity, Adolescents, School, Randomised controlled trial, Implementation, Multi-component, Scale-up

## Abstract

**Background:**

‘Physical Activity 4 Everyone’ (PA4E1) was an efficacious multi-component school-based physical activity (PA) program targeting adolescents. PA4E1 has seven PA practices. It is essential to scale-up, evaluate effectiveness and assess implementation of such programs. Therefore, the aim is to assess the impact of implementation support on school practice uptake of the PA4E1 program at 12 and 24 months.

**Methods:**

A cluster randomised controlled trial, utilising a type III hybrid implementation-effectiveness design, was conducted in 49 randomly selected disadvantaged Australian Government and Catholic secondary schools. A blinded statistician randomly allocated schools to a usual practice control (*n* = 25) or the PA4E1 program group (*n* = 24), with the latter receiving seven implementation support strategies to support school PA practice uptake of the seven practices retained from the efficacy trial. The primary outcome was the proportion of schools adopting at least four of the seven practices, assessed via telephone surveys with Head Physical Education Teachers and analysed using exact logistic regression modelling. This paper reports the 12-month outcomes.

**Results:**

Schools were recruited from May to November 2017. At baseline, no schools implemented four of the seven practices. At 12 months significantly more schools in the program group had implemented four of the seven practices (16/24, 66.7%) than the control group (1/25, 4%) (OR = 33.0[4.15–1556.4], *p* < 0.001). The program group implemented on average 3.2 (2.5–3.9) more practices than the control group (*p* < 0.001, mean 3.9 (SD 1.5) vs 0.7 (1.0)). Fidelity and reach of the implementation support intervention were high (both > 80%).

**Conclusions:**

Through the application of multiple implementation support strategies, secondary schools were able to overcome commonly known barriers to implement evidence based school PA practices. As such practices have been shown to result in an increase in adolescent PA and improvements in weight status, policy makers and practitioners responsible for advocating PA in schools should consider this implementation approach more broadly when working with schools. Follow-up is required to determine whether practice implementation is sustained.

**Trial registration:**

Australian New Zealand Clinical Trials Registry ACTRN12617000681358 registered 12th May 2017.

## Background

Despite unequivocal evidence of health benefits of physical activity (PA), pooled data from 298 school-based surveys from 146 countries indicates that globally, 81% of adolescents are insufficiently active [1]. PA typically declines 7% per year during adolescence [2]. As schools provide sustained access to adolescents [3], the World Health Organization and governments internationally have recommended the implementation of school-based policies, practices and programs that support adolescents to be physically active [4]. However, there is limited evidence that school-based programs targeting adolescents can impact on whole day PA [5, 6]. Systematic reviews and meta-analyses of school-based PA interventions in increasing device-assessed moderate-to-vigorous physical activity (MVPA) concluded such interventions have not been effective [5, 6]. The complex and multicomponent nature of most school-based PA interventions may make such interventions particularly vulnerable to poor implementation [7]. As such, further attention should be given to the application of implementation science theory and framework, and implementation fidelity [5, 6].

In one review, our cluster randomised controlled trial ‘Physical Activity 4 Everyone (PA4E1)’, conducted in 10 secondary schools in socio-economically disadvantaged areas of Australia [8], was one of only three identified effective school-based interventions targeting adolescents that demonstrated an effect on whole day PA [6]. Following the 24-month intervention period, adolescents attending schools allocated to the PA4E1 program were found to participate in 49 min more MVPA per week than those attending control schools [8], in addition to demonstrating a smaller increase in unhealthy weight gain over 2 years [9]. The PA4E1 program delivered these outcomes at a low incremental cost [10]. The multi-component program consisted of seven PA promoting practices: 1) increased PA within physical education (PE), 2) development of student personal PA plans, 3) enhanced school sport programs, 4) recess and lunchtime PA, 5) school PA policy, 6) links with community PA providers and 7) links with parents [8]. Schools were offered six support strategies to help them implement the seven practices, with four of the five program schools having implemented all PA practices, and the remaining school implementing six practices after 24-months [8]. The support strategies included a school change agent (PA consultant) 1 day per week, staff development and training for Physical Education teachers, schools establishing committees to oversee the changes, feedback reports to schools on progress towards practice adoption at the end of each term, email prompts to PE teachers, and provision of PA equipment.

Despite the development of effective programs such as PA4E1 [8, 10], unless such programs are implemented at-scale, the benefits at a population level are limited [11]. However, implementation, particularly implementation at-scale, remains a challenge [12, 13]. A Cochrane review of school-based implementation trials (described as the use of strategies to adopt and integrate evidence-based health interventions and to change practice patterns), included 27 studies, of which three tested multi-component strategies targeting the implementation of PA policies or practices in middle or secondary schools [13]. The review found most trials were not scaled up (*n* = 23) and concluded that implementation focused primarily on education strategies. As a result, the impact of strategies on policy and practice implementation was equivocal [13].

This trial is the first implementation trial targeting adolescent PA within secondary schools conducted within the Australian setting, and one of only three such trials internationally within middle or secondary schools [13]. The aim is to assess the effectiveness of implementation support strategies on implementation of the seven PA4E1 PA promoting practices within a larger number of secondary schools than the original efficacy trial, compared to usual school practice, over 12 and 24 months. The implementation support consisted of seven strategies based on the support provided in the efficacy trial, then adapted for scale-up based on the Theoretical Domains Framework (TDF) and the Behaviour Change Wheel (BCW). We hypothesise that the theoretically designed implementation support will result in greater PA practice uptake in secondary schools, compared to usual practice. This paper reports the 12-month school practice outcomes.

## Methods

### Study design

**Fig. 1:**
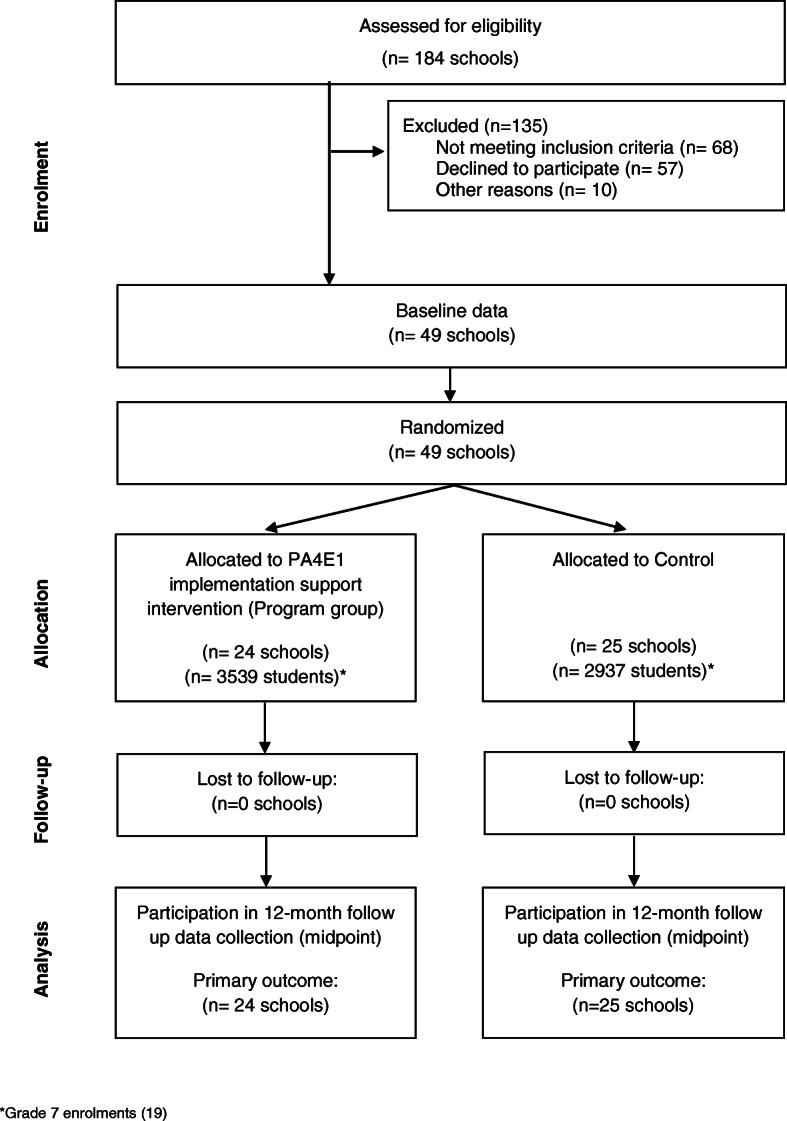
Consort Flow Diagram

A cluster randomised controlled trial was conducted with 49 secondary schools across four local health districts in New South Wales (NSW), Australia (Fig. 1). These districts include approximately 34% of Government and Catholic secondary schools, and 34% of the secondary school student population in NSW [14, 15]. The trial was a type III hybrid implementation-effectiveness trial combining both school-level implementation outcomes at 12 (reported in this paper) and 24 months, and individual student level PA and anthropometric outcomes at 12 and 24 months [16]. The primary trial outcome was the proportion of schools adopting at least four of the seven PA practices, assessed via telephone surveys with Head PE Teachers at baseline and follow-ups. Secondary outcomes included the mean number of PA practices implemented and the proportion of schools implementing each practice. The trial methods have been reported [16, 17]. The trial was prospectively registered ACTRN12617000681358 and approved by the Hunter New England Research Ethics Committee (Ref No. 11/03/16/4.05), University of Newcastle (Ref No. H-2011-0210), NSW Department of Education (SERAP 2011111), Maitland Newcastle Catholic School Diocese, Broken Bay Catholic School Diocese, Lismore Catholic School Diocese, Armidale Catholic School Diocese, and the Aboriginal Health and Medical Research Council. The trial adheres to the Consolidated Standards of Reporting Trials (CONSORT) Statement and checklist (Additional file 1) [18], the Standards for Reporting Implementation Studies (StaRI) Statement and checklist (Additional file 2) [19], and the TIDieR checklist (Additional file 3) [20].

### Participants

Schools were considered eligible if they: (i) were not intervention schools in the original PA4E1 trial [8]; (ii) were Government or Catholic schools; (iii) enrol students in Grades 7–9; (iv) were not specialist or fully selective/sports/performing arts/agriculture/boarding schools; (v) were located in areas classified as being disadvantaged by the SEIFA Index of Relative Socio-economic Disadvantage (suburb in lower 50% of NSW); (vi) were not participating in other major whole school PA trials or initiatives. School eligibility against ii-v was determined from publicly available data [14, 15, 21].

Recruitment of schools occurred from May to November 2017. A list of schools deemed eligible and within strata (based on local health district (four) and school sector (two) was assembled). Letters explaining the study were sent via email to the principals, requesting the information be shared with the Head PE Teacher. The Principal and/ or Head PE Teacher were contacted by telephone by a Project Officer who had a training background as a PE teacher, to invite their school to participate. A face-to-face or telephone meeting was offered to outline the requirements of the study, confirm eligibility and gain active written or oral consent.

Following school consent, all PE staff were provided, via email, a study information letter. At baseline and 12 months, the Head PE Teacher within each participating school was invited to participate in a telephone survey, with invitations issued via email and followed up with telephone calls. When Head PE Teachers were unavailable, they were asked to nominate a delegate. Consent was implied based on completion of the survey.

### Randomisation and masking

Stratified block randomisation was used to allocate consenting schools to one of two groups in a 1:1 ratio. Separate random block sequences of sizes two and four were used within each of eight strata. The blocks were created through SAS version 9.3. Population of the blocks involved using a random number generator in Microsoft Excel to randomise the order of the schools, prior to pasting into their respective stratum block. This was conducted by a statistician not involved in recruitment and blinded during the randomization phase. Principals were notified by research staff of their school’s allocation to either the intervention (program) or usual care control following baseline collection of school practice measures (Head PE Teacher surveys).

This was an open trial due to the inability to blind schools and teachers to the program strategies. Telephone interviewers were not informed of group allocation. The statistician undertaking the randomization and analysis was blinded to study group.

### Procedures

In both arms, baseline data were collected August–October 2017 and 12-month follow-up data from September–December 2018.

#### Program group (intervention)

The evidence-based PA4E1 program consisted of seven PA practices and has previously demonstrated a positive effect on student PA [8] and weight status [9]. The Health Promoting Schools Framework (consisting of three domains including i) Curriculum, teaching and learning, ii) Ethos and environment and iii) partnerships and services) and Social Ecological Theory which emphasizes the multiple layers of influence from individual, organisational, social and policy, underpinned the seven school PA practices used in the initial and current trial (Table 1) [8, 16]. A set of essential criteria was developed for each PA practice and schools were required to meet these elements in order to demonstrate implementation in the study outcome assessment. Four practices also contained additional desirable elements considered to improve implementation quality, which program schools were encouraged to meet.

**Table 1:** Overview of the evidence-based PA4E1 program (physical activity practices) including standards required of program schools (essential elements) and additional desirable elements [16]

***Physical activity practices by Health Promoting Schools domain***	
*Curriculum, teaching and learning*	
1. **Quality Physical Education (PE) lessons:**	
• PE department used documented principles or guidelines for teachers to maximize PE quality, active learning time and student engagement in PE lessons (Program schools used the SAAFE principles- Supportive, Autonomous, Active, Fair, Enjoyable) [22].	
• Each PE teacher participated in peer observation of a practical PE lesson, at least once a year^a^.	
• *Desirable – peer observation feedback is against the department’s quality PE principles.*	
2. **Student physical activity plans:**	
• All Grade 7 students developed a personal PA plan which included	
i) personal goals to improve or maintain activity or fitness	
ii) actions and timelines to achieve goals and	
iii) progress monitoring	
• Goals reviewed once within year	
• *Desirable – students in Grades 7–10 develop a personal PA plan*^b^	
3. **Enhanced school sport program:**	
• The school delivered a short (10–12 weeks) structured PA Program designed to improve adolescents’ fitness and provide them with knowledge, motivation and skills to engage in a range of lifelong physical activities.	
• The program should be delivered to all students in at least one Grade between 7 and 10 (Program schools delivered the Resistance Training for Teens program to all of Grade 7 [23].	
*Ethos and environment*	
4. **Recess/ lunchtime physical activity:**	
• Supervised recess and/or lunchtime PA sessions offered to all students in Grades 7–10 at least 3 days per week.	
• PA equipment freely available to students at least 3 days per week at recess and/or lunch.	
• *Desirable - at least one organized recess or lunch activity per week targeting girls. Sessions promoted to students at least once per term.*	
5. **School PA Policy or Procedure:**	
• School developed a policy which included:	
i) Provision of at least 150 min/week of MVPA during school time for all students in Grade 7–10;	
ii) Supportive practices to enhance all students’ PA (at least 3 of practices 1–4, 6–7 in this table)^c^	
*Partnerships and services*	
6. **Links with community physical activity providers:**	
• School has at least three links that went beyond promotion of the provider (e.g. in newsletters) to involve an agreement, connection, partnership (e.g. out of hours sessions on school facilities, presentation by providers at school).	
• Links were designed to support ‘outside of school time’ activity.	
• Links were communicated to students and families at least once per term^d^.	
• *Desirable - at least one of the community links made were to promote free or low cost options in the community.*	
7. **Communicating physical activity messages to all parents**	
• All parents of students in Grades 7–10 were sent PA messages that were designed to increase parent knowledge, attitudes and support towards PA, at least once per term (excludes messages about school events e.g. carnivals, or school sports timetables or results, or advertisements for community PA providers).^d^	

^a^Program schools were asked to aim for peer observations once a semester

^b^Program schools asked to set plans for Grade 7 at 12 months, Grades 7–10 for 24 months

^c^Program schools asked to include practices 1–4, 6 and 7 in their policy

^d^Program schools asked to use multiple modes to promote community links and to communicate PA information to parents (e.g. newsletters, parent app, parent information evening)

##### Implementation intervention

The Behaviour Change Wheel (BCW) [24] and the Theoretical Domains Framework (TDF) [25] were used to develop the implementation support strategies as described in the trial protocol [16]. The TDF brings together 33 models of behaviour change into 11 theoretical domains that explain the potential determinants of behaviour. The BCW was then used to link the identified behaviours to the mapping of appropriate behaviour change techniques (BCTs). This process resulted in seven overarching strategies, within which there were 23 sub-strategies (Table 2). Implementation support was designed to be delivered over eight school terms (two school years), and this paper reports the 12-month implementation outcomes following four school terms. Within this period one school term was dedicated to supporting school planning (Term 4, 2017), followed by three terms of supporting implementation of the PA practices with a focus on incoming Grade 7 students (age 12–13, first year of secondary school) (Term 1–3, 2018).

**Table 2:** Overview of the implementation support strategies for first 12 months (4 school terms)

***Implementation support strategies (n = 7) and sub-strategies (n = 23) (implemented over 4 school terms)***	***Fidelity (provided)*** *(n/N schools)*	***Reach (uptake)*** *(n/N schools)*
**1. Executive and leadership support**		
**1.1:** PA4E1 Partnership agreement signed by school executive.	N/A	24/24
**1.2:** New or existing school committee formed to oversee program.	N/A	15/24
**1.3:** The School committee is inclusive of in-School Champion and school executive to oversee the program.	N/A	10/24
**1.4:** Committee met at least once per term.	N/A	10/24
**2. Embedded school staff: in-School Champion**		
**2.1:** An existing school PE teacher is allocated the role of in-School Champion to support implementation for full 12 months.	N/A	24/24
**2.2:** The position was funded by the NSW Department of Health, half day per week (equivalent to $350AUD a fortnight).	24/24	24/24
**3. External implementation support**		
**3.1:** Health Promotion Support Officer (ideally a trained PE teacher) appointed to support schools with the program.	24/24	24/24
**3.2:** Health Promotion Support Officer was co-located within the relevant local health district.	17/24	17/24
**3.3:** Weekly contact was made with in-School Champion via phone, email and/or face-to-face site visits for 12 months.	N/A	N/A
**3.4:** Support Officer and in-School Champion have a face-to-face contact at least once a term.	24/24	18/24
**4. Teacher professional learning**		
**4.1:** In-School Champion training −1-day of face to face training session was hosted by PA4E1 implementation team in Term 1. Accommodation, meals and transport costs were covered by the NSW Department of Health.	24/24	24/24
**4.2:** Quality PE training for all PE teachers - 6 × 10-min online training videos followed by knowledge check short quizzes focused on the SAAFE principles were delivered via a password protected program website.	24/24	12/24
**4.3:** Enhanced school sport training – in-School Champions and other teachers involved in delivering the program could attend an existing 1 day face-to-face Resistance Training for Teens workshop offered by the NSW Department of Education (School Sport Unit), or equivalent training run by PA4E1 implementation team (not accredited). Course costs to be paid by project for in-School Champion, but not for other teachers.	24/24	23/24
**4.4:** School PA policy training – in-School Champion offered existing online training run by the NSW Department of Education School Sport Unit (Government schools only, *n* = 19) [26].	19/19	4/19
**5. Resources**		
**5.1:** Printed posters outlining Quality PE principles (SAAFE Principles [22]) to be displayed in PE department delivered to in-School Champions.	24/24	24/24
**5.2:** A $100AUD PA equipment voucher was provided to support the delivery of recess and lunchtime PA.	24/24	24/24
**5.3:** Equipment provided to support the delivery of recess and lunchtime PA enhanced schools sport program (5 Gymsticks/school)	24/24	22/24
**5.4:** Electronic resources housed on the program website (PA4E1 online) included: - overview of program presentation (Microsoft PowerPoint presentation) - project milestones to be achieved each term (over 4 terms) - online quality PE training (SAAFE Principle videos (6 videos - one overview and one per Principle) and worksheet, peer observation materials) - student personal PA plan templates - recess and lunch resources - policy templates - examples of community PA providers - tips and frequently asked questions	24/24	24/24
**6. Provision of prompts and reminders**		
**6.1:** Weekly emails or phone calls were made by the Support Officer to in-School Champions to encourage implementation.	16/24	16/24
**6.2:** Automated messages were sent each term via the program website to in-School Champions to prompt completion of teacher professional learning and online termly performance monitoring and feedback surveys.	24/24	24/24
**7. Implementation performance monitoring and feedback**		
**7.1**: In-School Champion completes all termly surveys via the program website (PA4E1 Online).	24/24	24/24
**7.2**: A feedback report is automatically generated and sent to in-School Champions via email	24/24	24/24
**7.3**: A feedback report is automatically generated and sent to school Principals via email.	24/24	6/24
**Total (%)**	**95.6**	**81.5**

Total fidelity and reach scores are percentage across all schools. Only sub-strategies with available data were included. Sub-totals for fidelity and reach percentages were calculated within each implementation strategy first, then sub-totals were averaged from all seven strategies to produce a final fidelity and reach percentage

*N/A* Excluded as there was no available data

#### Control group

The control schools continued with their usual practices and received no contact from the research team other than to organise data collection.

### Outcomes

The primary outcome was the proportion of schools in each group implementing any four of the seven PA practices. Our prior trial demonstrated a significant effect on student MVPA at 12-months when implementing four of the seven PA practices was achieved by program schools [27]. Secondary outcomes relating to school PA practices were: (i) the mean number of practices achieved; (ii) whether or not schools implemented each of the seven practices.

Measures of the school practices were undertaken via computer-assisted telephone interview surveys with Head PE Teachers, administered by trained interviewers. An overview of the questions, which asked about school practices within the current school year, and the responses required for a school to be considered to be meeting the practice, are shown in Additional file 4. The survey items were pilot tested and forwarded to participants prior to the interview. Self-report by school personnel is a feasible option for assessing school practices and has previously been demonstrated as a valid and reliable assessment of school PA practices [28, 29]. For program schools, Head PE Teachers were asked to speak with the teacher taking the role of PA4E1 in-School Champion prior to the survey.

Publicly available data provided information for all schools approached on school sector, postcode, size (total enrolments), Indigenous enrolments, and students who speak a language other than English at home [14, 15, 21]. For schools participating in the trial, the following characteristics were obtained through the Head PE teacher survey: number of PE teachers and full-time equivalent PE positions at the school; sex of Head PE Teacher, PE training, years of teaching experience, and how long they’ve taught PE at their current school.

### Process evaluation

A mixed methods process evaluation will be published separately as per the detailed process evaluation protocol, including assessment of modifications, fidelity, reach, acceptability, appropriateness and feasibility [17]. The current paper reports on the fidelity and reach of the seven implementation support strategies (within which there are 23 sub-strategies, see Table 2) [16, 17, 30]. Fidelity was operationalised as the percentage of implementation support strategies provided or offered to schools as prescribed in the original protocol. Reach was operationalised as the percentage of uptake of the implementation support strategies by schools [16, 17, 30] (see footnote Table 2). Data to assess the fidelity and reach of the implementation support strategies were obtained from the following sources: website usage data, in-School Champion implementation performance monitoring and feedback surveys completed once per term by program schools on the program website, and the Head PE Teacher survey.

### Economic and effectiveness evaluation

Both economic and effectiveness evaluation (device-measured student PA) will be published separately using methods detailed in the trial protocol [16].

### Statistical analyses

Analyses were conducted using SAS, version 9.3, from February–June 2019. Characteristics of schools participating in the trial and those refusing were compared using Chi-square analyses. School characteristics were summarised for program and control schools. Analysis followed intention to treat principles, where schools were analysed according to their randomised treatment allocation. Given the low levels of practices at baseline, the small number of schools per group and almost no practice uptake by controls, the planned generalised linear regression models [16] were not considered appropriate for the dichotomous outcomes (implementing at least four of the seven practices (primary outcome) (yes, no), and each of the seven practices (secondary)) at 12 months. Instead, each dichotomous outcome was compared between groups using an exact logistic regression model adjusting for baseline outcome and for the stratification variables (LHD, school sector). For four practices, an additional model was undertaken based on whether schools met the desirable criteria (Table 1). For consistency in analysis approach the continuous secondary outcome variable (number of PA practices), a linear regression model was used to assess differences between groups at 12 months, adjusting for baseline and for the stratification variables. Significance levels for the analyses were set at *p* < 0.025 to allow for program effects at 12 or 24 months.

We estimated there would be approximately 120 eligible schools. Based on consent rates obtained previously (65–70%) [8], a sample of 76 schools (38 per arm) was estimated to provide 80% power to detect an absolute increase of ~ 35% between groups in the proportion of schools implementing at least four of the seven practices at 12 and 24-months. Without prior data on baseline levels of school practices, this calculation made the assumption that 40% of schools in the control arm could achieve this target at follow-up.

### Role of the funding source

The funder of the study had no role in study design, data collection, data analysis, data interpretation, or writing of the report. The corresponding author had full access to all the data in the study and had final responsibility for the decision to submit.

## Results

### Sample

There were 184 secondary schools assessed for eligibility (Fig. 1). Five schools were ineligible due to inclusion as a program school in the original PA4E1 trial. Publicly available data [14, 15, 21] excluded 52 schools (independent school (*n* = 8), senior school (*n* = 11), fully selective, Sports, Agricultural, Performing Arts, boarding schools (*n* = 7), suburb not in lower 50% of NSW for SEIFA (*n* = 26)). Eleven schools were excluded as they were involved in other PA /obesity studies or initiatives, seven schools were ineligible because the schools’ research governance body did not approve the study, and one due to sampling error. This left 108 schools sent study letters. Two further schools were subsequently excluded due to school restructures. Of the remaining 106 schools, 49 consented and 57 declined (Fig. 1, 46% consent rate). The reasons for declining (*n* = 88), where 25 schools gave more than one reason, were: lack of capacity to meet Support Strategy 2 (provide an in-School Champion (*n* = 61), lack capacity to meet Support Strategy 1 (executive support (*n* = 11), concerns about the intervention/research trial (allocation to control group, insufficient financed time for in-School Champion, feasibility of practice(s) (*n* = 6) and no reason/not interested (*n* = 10).

A comparison of characteristics (sector, local health district, remoteness, size, percentage enrolment of Indigenous student (< 10, 10% or more), percent enrolment of students of language other than English background (< 10, 10% or more)) for schools refusing versus consenting to participate showed no significant differences, except that schools refusing to participate were more likely to have 10% or more students of language other than English background (24/57 42% of refusers, 8/49 16% of consenting, *p* = < 0.05).

Characteristics of program and control schools, including respondent characteristics at baseline and 12 months, are shown in Table 3. Groups were similar, although there were more large schools and fewer medium schools in the Program group. For 38/49 schools, the telephone survey was completed by the same person at baseline and 12 months (18/24 program, 20/25 control). The teacher undertaking the baseline survey within 13 program schools took on the role of PA4E1 in-School Champion after baseline data collection, and 11 of these teachers also completed the interview at 12 months.

**Table 3:** Characteristics of program and control schools

***Characteristic***	***Program (n = 24)*** ***N***		***Control (n = 25)*** ***n***	
*Secondary school in eligible Local Health Districts*				
All eligible Local Health Districts	24		25	
- Central Coast	1		2	
- Hunter New England	12		12	
- Mid North Coast	5		6	
- South Western Sydney	3		4	
*School Sector*				
Government	19		21	
Catholic	5		4	
*School - SEIFA Index of Relative Socio-economic disadvantage (school suburb)*^a^				
Decile 1 (Most disadvantaged in State)	8		9	
Decile 2	7		3	
Decile 3	2		5	
Decile 4	6		6	
Decile 5	1		2	
*Remoteness*				
Major Cities	11		13	
Inner Regional	9		10	
Outer Regional/Remote	4		2	
*School size (total enrolments)*				
Small (< 400)	2		2	
Medium (400–800)	8		17	
Large (> 800)	14		6	
*Grade 7 size*				
Small (< 65)	3		3	
Medium (65–135)	7		14	
Large (> 135)	14		8	
**Total Students**	3539		2937	
*% female enrolments*				
< 30%	0		1	
30–40%	0		0	
41–50%	11		15	
51–60%	6		5	
100% (female only schools)	2		0	
*% Indigenous enrolments*				
< 10%	12		10	
> =10%	12		15	
- 10–24%	8		14	
- 25–49%	4		1	
*% Language Background Other Than English*				
< 10%	19		22	
> =10%	5		3	
- 10–24%	1		0	
- 25–49%	0		0	
- 50–74%	1		2	
- > =75%	3		1	
*PE Faculty size*				
1–4 FTE (3–12 staff)	5		6	
5–8 FTE (4–13 staff)	16		17	
9–13 FTE (9–15 staff)	3		2	
*Respondent (Head PE teacher or delegate)*	*Baseline*	*12 months*	*Baseline*	*12 months*
**Gender**				
- Male	16	14	16	15
- Female	8	10	9	10
**PE trained**				
- Yes	24	24	25	25
**Years of teaching experience**				
- < 1	0	0	1	0
- 1 to 5	3	1	0	0
- 6 to 10	6	7	4	7
- 11 to 15	1	4	6	7
- 16 to 20	5	0	5	0
- > 21	9	8	10	7
**Years teaching PE at current school**				
- New in that year	2	1	2	1
- < 3	0	3	2	3
- 3 to 5	4	3	3	3
- > 5	18	17	18	18

^a^ Schools in deciles 6–10 were not eligible for the trial

### School practice outcomes

Table 4 presents the school practice results.

**Table 4:** School implementation of physical activity practices at baseline and 12-month follow-up

School practice implementation in current school year	Program Group		Control Group		OR [95% CI]	***P*** value
	Baseline *N* = 24% (n)	12 months *N* = 24% (n)	Baseline *N* = 25^a^ % (n)	12 months *N* = 25% (n)		
**Primary outcome** – 4 or more PA practices	0.0 (0)	66.7. (16)	0.0 (0)	4.0 (1)	***35.5 [4.5–1659.6]***	***< 0.001***
**Secondary outcomes**						
Mean number of PA practices (standard deviation)	0.5 (0.8)	3.9(1.5)	0.5(0.7)	0.7 (1.0)	***β = 3.2 [2.5–3.9]***^***b***^	***< 0.001***
Meeting each PA practice						
1. Quality PE lessons	8.3(2)	58.3(14)	4.0 (1)	4.0 (1)	33.5 [3.9–1654.3]	***< 0.001***
*Incorporating desirable elements (*Table 1*)*	8.3(2)	54.2(13)	4.0(1)	4.0(1)	29.5 [5.7 -∞]	***< 0.001***
2. Student physical activity plans	8.3 (2)	87.5 (21)	0.0 (0)	8.0 (2)	79.7 [9.0–4159.9]	***< 0.001***
*Incorporating desirable elements (*Table 1*)*	0.0 (0)	8.3 (2)	0.0 (0)	4.0 (1)	1.7 [0.1–106.1]	*1.0*
3. Enhanced school sport program	0.0 (0)	83.3 (20)	4.0 (1)	4.0 (1)	86.0 [16.5-∞]	***< 0.001***
4. Recess/lunchtime physical activity	8.3 (2)	50.0 (12)	24.0 (6)	28.0 (7)	4.1 [1.0–22.6]	0.059
*Incorporating desirable elements (*Table 1*)*	*0.0 (0)*	*33.3 (8)*	*8.0 (2)*	*8.0 (2)*	10.0 [1.2–473.4]	0.027
5. Physical activity policy or procedure	0.0 (0)	29.7 (7)	4.0 (1)	4.0 (1)	9.8 [1.1–492.3]	0.044
6. Links with community physical activity providers	4.1 (1)	0.0 (0)	0.0 (0)	4.0 (1)	1.0 [0.0–19.0]	1.0
*Incorporating desirable elements (*Table 1*)*	*4.1 (1)*	*0.0 (0)*	*0.0 (0)*	*4.0 (1)*	1.0 [0.0–19.0]	1.0
7. Communicating physical activity messages to parents	20.8 (5)	83.3 (20)	a 16.0 (4)	20.0 (5)	16.3 [3.4–120.2]	***< 0.001***

^a^ there were two schools with missing parent practice data in the control group at baseline. These schools were included in the baseline analysis for the primary outcome as ‘not met’ they both had one practice, and an additional practice would not have enabled them to meet the four practice criteria. Analyses based on mean number of practices, and the parent practice, are similarly based on the practice being ‘not met’. When analyses were based on a reduced sample due to the missing data the results were consistent: baseline N, mean practices (SD) 23 0.9 (0.81) *p* < 0.001; parent practice N % (n) 23 17.4 (4) *p* < 0.001

^b^ Mean difference estimate from linear regression analysis

#### Primary outcome

*Proportion of schools adopting at least four of the seven PA practices*: Due to the low fraction of missing data (two schools missing data for one practice at baseline), missing baseline data was assumed to be ‘not implementing’ (sensitivity analyses counting the data as missing, see Table 4 footnote). At baseline, no schools had implemented four of the seven practices in the current school year. At 12 month follow-up, significantly more schools had implemented four of the seven practices in the program group (17/24, 70.8%) than the control group (1/25, 4%) (*p* < 0.001).

#### Secondary outcomes

*i) Mean number of practices achieved*: After adjusting for baseline differences, the program group was implementing on average 3.2 (2.4–3.9) more practices than the control group at 12 months (*p* < 0.001, mean 3.9 (SD 1.5) vs 0.7 (SD 1.0)). *(ii) whether or not schools implemented each of the seven practices*: The program group achieved a significantly (*p* < 0.025) higher proportion of schools implementing the following four PA practices – quality PE lessons, student PA plans, enhanced school sport program, and providing PA messages to parents. For the remaining three PA practices, the differences at 12 months were not statistically significant: school PA policy (*p* = 0.044), recess/lunchtime PA (*p* = 0.059) and establishing links between the school and community PA providers (*p* = 1.00).

### Process evaluation

As per the methods, a separate detailed process evaluation will be published [17]. Table 2 shows the fidelity and reach data for implementation support strategies over the 12 months. The overall mean fidelity score across all schools was 95.6% (SD 6.4), range 86.1–100%, median 100%. The overall mean reach of the implementation strategies across all schools was 81.5% (SD 7.5), range 69.0–91.7%, median 82.1%, indicating that schools received the majority of sub-strategies (Table 2).

## Discussion

This is the first cluster randomised controlled trial to assess the effectiveness of a multi-component implementation support strategy on improving implementation by schools, at-scale, of evidence-based PA promoting practices within socio-economically disadvantaged secondary schools. After 12 months, the proportion of schools implementing the required PA practices had significantly improved, relative to the control schools, with more than 70% of secondary schools implementing at least four of the seven PA4E1 practices. Schools in the program group were implementing on average 3.2 more school PA practices than schools in the control group. For six of the seven practices uptake improved after 12 months, and for four of these practices the proportion of program and control schools implementing the practice at 12 months was statistically significant. Through the development of a multi-component implementation support intervention delivered with high fidelity and reach, the level of PA practice implementation appears comparable to the original effectiveness trial conducted with ten secondary schools [8]. Within this original trial, program schools were supported to implement four practices within the first 12 months (active PE, student PA plans, recess/lunch physical activities, and PA messages to parents), with all five schools implementing these practices. With very few school-based PA interventions having been scaled up [12], and research demonstrating that interventions that have been scaled-up often have a reduced effect due to poor implementation [31], the 12-month results appear promising. Longer-term follow up is needed to determine if this level of practice change is able to be increased or sustained in the subsequent 12-month phase of implementation support [8]. The effectiveness of the intervention at 12-month follow-up appears large relative to previous trials included within a Cochrane review of school-based implementation trials, which found increases in implementation of less than 20% [13]. These findings support the use of a multi-component implementation support model to increase schools implementation of evidence-based PA practices.

Whilst the intervention demonstrated large improvements in the proportion of schools implementing at least four of the seven practices, the practices were not adopted equally. There was a significantly higher proportion of schools in the program group implementing the curriculum-related practices including quality PE lessons, student PA plans and enhanced school sport programs. In addition, a greater proportion of schools in the program group were communicating PA messages to parents compared to those in the control. This is consistent with literature indicating that implementation of curriculum-based strategies appears more feasible and acceptable than school environment and policy-related practices [13]. A well-developed implementation support strategy delivered with high fidelity and reach is required to attain changes in policy and school environment outcomes [13, 32]. Teachers’ report lack of time, self-efficacy and specific training as the major barriers to implementing whole-of-school PA practices [32, 33]. Implementation support models which address such barriers, including providing short, practical and online training that can be completed amongst other competing teaching demands in addition to observation, feedback and technical support may be key in supporting implementation of broader school environment strategies. School-based PA interventions are particularly vulnerable to poor implementation (fidelity/reach) due to their complex and multicomponent nature [7]. Our implementation support intervention (the seven implementation support strategies) were delivered with high fidelity and reach (both > 80%).

Whilst there was a trend towards higher implementation of two other practices, development of a school PA policy (*p* = 0.044) and recess/lunchtime PA (*p* = 0.059) in comparison to the control group, the final practice of linking to the broader community to provide outside of school PA opportunities did not differ between groups. This indicates that PA practices related to changing the school environment appear more challenging to implement in comparison to school curriculum practices; however, show promise that at least some of these practices may be feasible to implement. It is unclear if further implementation support is warranted or if schools require additional time to enable implementation to occur [8, 34]. The implementation of strategies beyond the school environment appears to be an ongoing challenge [34], which is also consistent with other school-based interventions reporting low practice uptake [34]. Nonetheless, systematic reviews of PA interventions demonstrate that those which include the implementation of practices linking with the community are more likely to result in an effect on student PA levels [35]. Identifying barriers and suitable strategies to support implementation of such strategies remains important.

The strengths of this study include the randomised controlled study design and the inclusion of a support intervention designed using theoretically-informed implementation frameworks [13]. Despite these strengths, a number of limitations should be noted. Whilst self-reported practice outcomes have been reported as valid and reliable, self-reported measures are subjective in nature and may be subject to response bias [29]. Social desirability effects in program schools cannot be discounted, perhaps in particular for schools in which the teacher taking on the in-School Champion role provided the survey data. Head PE teachers in these schools nominated the in-School Champion as it was perceived they would be best able to provide detailed responses. To overcome these limitations, the definition of each practice was agreed apriori by the advisory group and implementation team, based on the previous efficacy trial and evidence in the literature of supporting change in PA levels of adolescents. For two practices where inconsistent interpretation of the question seemed likely (set of quality PE principles, running an enhanced school sport program), decisions about whether schools met the practice included rating of responses by PE teachers within the research organisation (Additional file 4).

The eligibility and consent rates should also be noted, with just under half of Government and Catholic schools (main school providers in NSW) across the study region eligible due to targeting schools located in lower socio-economic regions. As a result, we are unsure how the results may apply to non-eligible school types such as those located in higher socioeconomic areas and specialist schools such as those targeting academic or performing arts students. Further, the consent rate was lower than anticipated at 46%, indicating that some schools were reluctant to participate and the intended sample size of 76 schools was not obtained. However, based on baseline data collection and number of schools recruited, a revised power calculation assuming practice adoption in 10% of control schools at follow-up (rather than 40%), still allowed detection of a 35% difference between groups at follow-up. There is potential the lower than anticipated consent may be due to the research nature and additional measurement burden placed on schools. However, we are unsure if participation would differ if the program was not offered as a research study.

## Conclusion

The 12-month outcomes of the PA4E1 implementation trial provide promising evidence on the effectiveness of strategies to support the scale-up of effective school-based PA programs. The multi-component implementation support intervention, which was underpinned by theory, resulted in a significant increase in the number of PA practices implemented by secondary schools. Ongoing implementation and evaluation at 24-months will determine whether the same implementation support over a longer duration will maintain, increase or decrease the number of practices schools implement and whether practices that were harder to shift over 12-months can be implemented given longer support. The results suggest policy makers and health promotion practitioners responsible for advocating for PA in schools should consider this implementation approach. Our forthcoming mixed methods process evaluation will provide additional information on how school stakeholders perceived and received the support – allowing potential adjustment of the support model for future application.

## Supplementary information

**Additional file 1.** Consort Checklist.

**Additional file 2.** StaRI Checklist.

**Additional file 3.** TIDieR Checklist.

**Additional file 4.** Overview of the interview questions assessing school physical activity practice implementation.

## Data Availability

All study materials are available from the research team upon request to lead investigators. All data will be stored securely as per ethical requirements. All participants will be issued a unique identification number following consent for confidentiality. The final trial dataset will be stored securely and accessed only by the study statistician. The results of this trial will be disseminated via publication in a peer-reviewed journal, conference presentations, plain language summaries and reports to schools and relevant health and education departments.

## Abbreviations

PA: Physical activity
PA4E1: Physical Activity 4 Everyone
MVPA: Moderate-to-vigorous intensity physical activity
NSW: New South Wales
PE: Physical education
